# Supplementary material for: Pediatric Clinical Classification System for use in Canadian inpatient settings
Source: PLoS One. 2022 Aug 25;17(8):e0273580. doi: 10.1371/journal.pone.0273580 (PMC9409563; doi:10.1371/journal.pone.0273580)
Supplement: Attachment — Submitted filename: Review_PONE-D-22-13110.docx [file pone.0273580.s002.docx]

Abstract:

- In the background section, it would be helpful for a broader audience if you add some commentary on why a classification system for organizing diagnostic codes is needed- (e.g., in order to enable analyses of administrative datasets that answer important clinical and health services questions). You comment on this in the conclusion, but the rationale for conversion might be more helpful right at the beginning.

Introduction:

- Again, would be helpful to take some space here to explain that one cannot meaningfully analyze administrative datasets without such groupers because analyses cannot accommodate/run when several thousand diagnosis codes are included. By grouping them, we are able to ask important research questions using advanced observational study designs.
- It would also be helpful to provide some brief literature review here of examples of using PECCS or CCS to define high-priority conditions or answer helpful clinical research questions.
- Need to add some background on the other classification system options available and their limitations- why do we need PECCS-CA?

Methods:

- Would be helpful to provide brief descriptions of the data sources and elements contained in each as well as how they are linked and how data quality is monitored/maintained

Results

- The top 10 rankings seem a bit surprising and different from other prior similar analyses. Although this may be out of the scope of this analysis, it would be helpful to directly compare the results of using this grouper PECCS-CA vs CCS using the same dataset, or at least qualitatively compare the findings to such prior analyses. That would allow some more in depth assessment of the validity/success of the authors mapping process as well as potentially enable authors to directly demonstrate the advantages of this new classification system. Those would both be valuable additions to both the results and discussion.
