# Supplementary material for: Pediatric Clinical Classification System for use in Canadian inpatient settings
Source: PLoS One. 2022 Aug 25;17(8):e0273580. doi: 10.1371/journal.pone.0273580 (PMC9409563; doi:10.1371/journal.pone.0273580)
Supplement: Attachment — Submitted filename: Response to Reviewers.docx [file pone.0273580.s003.docx]

July 14, 2022

Jiangtao Gou, PhD

Academic Editor, Public Library of Science (PLOS) ONE

Villanova, PA 19085, United States

**RE: Manuscript – Pediatric Clinical Classification System for use in Canadian inpatient settings**

Dear Dr. Gou,

Thank you for considering our research article submitted to the *PLOS ONE*. Currently, we have published one related paper that used the PECCS-CA to classify ICD-10-CA discharge diagnosis codes of inpatient encounters into discrete clinical condition categories in the *JAMA Network Open* journal (Gill PJ *et al.* JAMA Netw Open 2022;5:e2147447). Other than this paper, we have not published or submitted any other related papers from this study.

We have reviewed the comments provided by the academic editor and the reviewer(s), and we have attached a revised version of the manuscript. Please find below our response to each point raised by the academic editor and the reviewer(s). All authors of this manuscript agree with the revisions outlined in this letter and in the attached revised version of the manuscript.

**JOURNAL REQUIREMENTS**

When submitting your revision, we need you to address these additional requirements.

**JR1.1** Please ensure that your manuscript meets PLOS ONE's style requirements, including those for file naming. The PLOS ONE style templates can be found at 
[https://journals.plos.org/plosone/s/file?id=wjVg/PLOSOne_formatting_sample_main_body.pdf](https://urldefense.com/v3/__https:/journals.plos.org/plosone/s/file?id=wjVg*PLOSOne_formatting_sample_main_body.pdf__;Lw!!D0zGoin7BXfl!-MRa53UALKDUf89OUVuV6G6Wm5BCskJ4Gumovk4h853FnskRx-Xi-5elo572tEv_wYoAdm9Q0aAOoAlPDbMPZXBn$) and 
[https://journals.plos.org/plosone/s/file?id=ba62/PLOSOne_formatting_sample_title_authors_affiliations.pdf](https://urldefense.com/v3/__https:/journals.plos.org/plosone/s/file?id=ba62*PLOSOne_formatting_sample_title_authors_affiliations.pdf__;Lw!!D0zGoin7BXfl!-MRa53UALKDUf89OUVuV6G6Wm5BCskJ4Gumovk4h853FnskRx-Xi-5elo572tEv_wYoAdm9Q0aAOoAlPDRh9UAtz$)

Response: We have ensured that our manuscript meets PLOS ONE’s style requirements.

**JR1.2** We note that the grant information you provided in the ‘Funding Information’ and ‘Financial Disclosure’ sections do not match.

When you resubmit, please ensure that you provide the correct grant numbers for the awards you received for your study in the ‘Funding Information’ section.

Response: We have ensured that the grant information provided in the ‘Funding Information’ section matches with the ‘Financial Disclosure’ section. We checked the ‘Funding Information’ section and the grant numbers do match with the ‘Financial Disclosure’ section. Please see the ‘Financial Disclosure’ presented below:

*“****Financial Disclosure:*** *This study was funded through the New Investigator grant (21-01) from the Physicians' Services Incorporated (PSI) Foundation. The authors who received the award were PJG, TT, and SM. The funders had no role in study design, data collection and analysis, decision to publish, or preparation of the manuscript.”*

**JR1.3** Thank you for stating the following in the Competing Interests section:

"We have read the journal's policy and the authors of this manuscript have the following competing interests:

PJG and SM have received grants from the Physicians' Services Incorporated (PSI) Foundation during the conduct of the study and grants from the Canadian Institute of Health Research (CIHR). PJG has also received personal fees from CIHR and EBMLive outside the submitted work. SM has also received personal fees from the Journal of Hospital Medicine outside the submitted work. The above indicated items does not alter our adherence to PLOS ONE policies on sharing data and materials."

Please confirm that this does not alter your adherence to all PLOS ONE policies on sharing data and materials, by including the following statement: "This does not alter our adherence to  PLOS ONE policies on sharing data and materials.” (as detailed online in our guide for authors [http://journals.plos.org/plosone/s/competing-interests](https://urldefense.com/v3/__http:/journals.plos.org/plosone/s/competing-interests__;!!D0zGoin7BXfl!-MRa53UALKDUf89OUVuV6G6Wm5BCskJ4Gumovk4h853FnskRx-Xi-5elo572tEv_wYoAdm9Q0aAOoAlPDZY_T9Uy$)).  If there are restrictions on sharing of data and/or materials, please state these. Please note that we cannot proceed with consideration of your article until this information has been declared.

Please include your updated Competing Interests statement in your cover letter; we will change the online submission form on your behalf.

Response: We have now revised the Competing Interests statement to include the above indicated statement. We have also added this updated Competing Interests statement into our cover letter and is indicated below:

***“Competing Interests Statement:*** *We have read the journal's policy and the authors of this manuscript have the following competing interests:*

*PJG and SM have received grants from the Physicians' Services Incorporated (PSI) Foundation during the conduct of the study and grants from the Canadian Institute of Health Research (CIHR). PJG has also received personal fees from CIHR and EBMLive outside the submitted work. SM has also received personal fees from the Journal of Hospital Medicine outside the submitted work. This does not alter our adherence to PLOS ONE policies on sharing data and materials.”*

**JR1.4** In your Data Availability statement, you have not specified where the minimal data set underlying the results described in your manuscript can be found. PLOS defines a study's minimal data set as the underlying data used to reach the conclusions drawn in the manuscript and any additional data required to replicate the reported study findings in their entirety. All PLOS journals require that the minimal data set be made fully available. For more information about our data policy, please see [http://journals.plos.org/plosone/s/data-availability](https://urldefense.com/v3/__http:/journals.plos.org/plosone/s/data-availability__;!!D0zGoin7BXfl!-MRa53UALKDUf89OUVuV6G6Wm5BCskJ4Gumovk4h853FnskRx-Xi-5elo572tEv_wYoAdm9Q0aAOoAlPDYAK7cRP$).

Upon re-submitting your revised manuscript, please upload your study’s minimal underlying data set as either Supporting Information files or to a stable, public repository and include the relevant URLs, DOIs, or accession numbers within your revised cover letter. For a list of acceptable repositories, please see [http://journals.plos.org/plosone/s/data-availability#loc-recommended-repositories](https://urldefense.com/v3/__http:/journals.plos.org/plosone/s/data-availability*loc-recommended-repositories__;Iw!!D0zGoin7BXfl!-MRa53UALKDUf89OUVuV6G6Wm5BCskJ4Gumovk4h853FnskRx-Xi-5elo572tEv_wYoAdm9Q0aAOoAlPDdz1hwy1$). Any potentially identifying patient information must be fully anonymized.

Important: If there are ethical or legal restrictions to sharing your data publicly, please explain these restrictions in detail. Please see our guidelines for more information on what we consider unacceptable restrictions to publicly sharing data: [http://journals.plos.org/plosone/s/data-availability#loc-unacceptable-data-access-restrictions](https://urldefense.com/v3/__http:/journals.plos.org/plosone/s/data-availability*loc-unacceptable-data-access-restrictions__;Iw!!D0zGoin7BXfl!-MRa53UALKDUf89OUVuV6G6Wm5BCskJ4Gumovk4h853FnskRx-Xi-5elo572tEv_wYoAdm9Q0aAOoAlPDX44adWJ$). Note that it is not acceptable for the authors to be the sole named individuals responsible for ensuring data access.

We will update your Data Availability statement to reflect the information you provide in your cover letter.

Response: We have revised the Data Availability statement following the above instructions. The data used in this study was obtained from ICES. The legal restrictions and data sharing agreements prohibits ICES from making the dataset publicly available, thus, we are unable to publicly share the data that were used in this study. However, readers who are interested in accessing the linked data can access it through the ICES Data & Analytic Services (DAS). Please see the data availability statement for this study indicated below:

***“Data Availability Statement:*** *The dataset for this study is held securely in coded form at ICES. The ICES is a prescribed entity under the Ontario’s Personal Health Information Privacy Act (PHIPA). As a prescribed entity, ICES is allowed to collect personally identifiable information for analysis that evaluate, plan, and/or monitor the health care system or for analysis related to the health or safety of the public without receiving individual consent. While legal data sharing agreements between ICES and data providers (e.g., healthcare organizations and government) prohibit ICES from making the dataset publicly available, access may be granted to those who meet pre-specified criteria for confidential access, available at www.ices.on.ca/DAS (email:* [*das@ices.on.ca*](mailto:das@ices.on.ca)*). Details on submitting a request form to access the data is found in the following hyperlink (*[*https://www.ices.on.ca/DAS/Submitting-your-request*](https://www.ices.on.ca/DAS/Submitting-your-request)*). The full dataset creation plan is available from the authors upon request.”*

**JR1.5** One of the noted authors is a group or consortium Canadian Paediatric Inpatient Research Network. In addition to naming the author group, please list the individual authors and affiliations within this group in the acknowledgments section of your manuscript. Please also indicate clearly a lead author for this group along with a contact email address.

Response: We have now listed the individual group members within the group, Canadian Paediatric Inpatient Research Network, in the Acknowledgements section of the manuscript. The group members listed below are non-author collaborators or contributors. This is similar to the non-author collaborators acknowledged in the following PLOS One article: (<https://journals.plos.org/plosone/article?id=10.1371/journal.pone.0230587#ack>). The affiliation for each individual member is removed in the Acknowledgements section of the manuscript but is indicated in this response below for completion. We have also added the lead author for this group along with their contact email address in this section of the manuscript, which is the same as the corresponding author for the manuscript (Acknowledgements, page 20, lines 348 - 351):

*“****Canadian Paediatric Inpatient Research Network (PIRN):*** *Patricia C Parkin MD^1,2,3,4^, Ann Bayliss MD^5^, Ronik Kanani MD^1,6^, Sean Murray MD^7^, Catherine Pound MD MSc^8,9^, Mahmoud Sakran MD^10,11^, Anupam Sehgal MD^10^, Sepi Taheri MD^12^, and Gita Wahi MD MSc^13^. The affiliations for the PIRN members include: ^1^Department of Pediatrics, University of Toronto, Toronto, Ontario, Canada; ^2^The Hospital for Sick Children, Toronto, Ontario, Canada; ^3^Child Health Evaluative Sciences, SickKids Research Institute, Toronto, Ontario, Canada; ^4^Institute of Health Policy, Management and Evaluation, Dalla Lana School of Public Health, The University of Toronto, Toronto, Ontario, Canada; ^5^Children’s Health Division, Trillium Health Partners, Mississauga, Ontario, Canada; ^6^Department of Pediatrics, North York General Hospital, Toronto, Ontario, Canada; ^7^Department of Pediatrics, Northern Ontario School of Medicine, Sudbury, Ontario, Canada; ^8^Children’s Hospital of Eastern Ontario, Ottawa, Ontario, Canada; ^9^University of Ottawa, Ottawa, Ontario, Canada; ^10^Department of Pediatrics, Queens University, Kingston, Ontario, Canada; ^11^Department of Pediatrics, Lakeridge Health, Oshawa, Ontario, Canada; ^12^Department of Pediatrics, Western University, London, Ontario, Canada; ^13^Division of General Pediatrics, Department of Pediatrics, McMaster University and McMaster Children’s Hospital, Hamilton, Ontario, Canada. The lead author for PIRN is Peter J Gill MD, DPhil, and his contact email address is* [*peter.gill@sickkids.ca*](mailto:peter.gill@sickkids.ca).”

**JR1.6** Your ethics statement should only appear in the Methods section of your manuscript. If your ethics statement is written in any section besides the Methods, please move it to the Methods section and delete it from any other section. Please ensure that your ethics statement is included in your manuscript, as the ethics statement entered into the online submission form will not be published alongside your manuscript.

Response: The ethics statement is in the Methods section of the manuscript. Please see subsection ‘Study design and data source’ under the ‘Methods’ section of the manuscript (page 8, lines 172 - 173).

**JR1.7** Please review your reference list to ensure that it is complete and correct. If you have cited papers that have been retracted, please include the rationale for doing so in the manuscript text, or remove these references and replace them with relevant current references. Any changes to the reference list should be mentioned in the rebuttal letter that accompanies your revised manuscript. If you need to cite a retracted article, indicate the article’s retracted status in the References list and also include a citation and full reference for the retraction notice.

Response: We have reviewed our reference list and revised a few of the references to ensure that the format of the references follows the Vancouver reference style as indicated in PLOS ONE’s submission guidelines webpage. We have tracked these changes on the manuscript.

**ADDITIONAL EDITOR COMMENTS**

This manuscript has been reviewed by two experts. Please follow their comments and revise your manuscript.

[Note: HTML markup is below. Please do not edit.]

**REVIEWERS’ COMMENTS**

**Reviewer's Responses to Questions**

**Comments to the Author**

**RC1.1** Is the manuscript technically sound, and do the data support the conclusions?

The manuscript must describe a technically sound piece of scientific research with data that supports the conclusions. Experiments must have been conducted rigorously, with appropriate controls, replication, and sample sizes. The conclusions must be drawn appropriately based on the data presented.

Reviewer #1: Yes

Reviewer #2: Yes

Response: Thank you for your response.

**RC1.2** Has the statistical analysis been performed appropriately and rigorously?

Reviewer #1: Yes

Reviewer #2: Yes

Response: Thank you for your response.

**RC1.3** Have the authors made all data underlying the findings in their manuscript fully available?

The PLOS Data policy requires authors to make all data underlying the findings described in their manuscript fully available without restriction, with rare exception (please refer to the Data Availability Statement in the manuscript PDF file). The data should be provided as part of the manuscript or its supporting information, or deposited to a public repository. For example, in addition to summary statistics, the data points behind means, medians and variance measures should be available. If there are restrictions on publicly sharing data—e.g. participant privacy or use of data from a third party—those must be specified.

Reviewer #1: Yes

Reviewer #2: No

Response: Thank you for your response. We have now revised the Data Availability statement for this study. The data used in this study was obtained from ICES. The legal restrictions and data sharing agreements prohibits ICES from making the dataset publicly available, thus, we are unable to publicly share the data that were used in this study. However, readers who are interested in accessing the linked data can access it through the ICES Data & Analytic Services (DAS). Please see the revised data availability statement indicated as part of the response to the journal requirement comment (**JR1.4**) indicated above.

**RC1.4** Is the manuscript presented in an intelligible fashion and written in standard English?

PLOS ONE does not copyedit accepted manuscripts, so the language in submitted articles must be clear, correct, and unambiguous. Any typographical or grammatical errors should be corrected at revision, so please note any specific errors here.

Reviewer #1: Yes

Reviewer #2: Yes

Response: Thank you for your response.

**RC1.5** Review Comments to the Author

Please use the space provided to explain your answers to the questions above. You may also include additional comments for the author, including concerns about dual publication, research ethics, or publication ethics. (Please upload your review as an attachment if it exceeds 20,000 characters)

***REVIEWER #1:***

**R1.1** Abstract - In the background section, it would be helpful for a broader audience if you add some commentary on why a classification system for organizing diagnostic codes is needed- (e.g., in order to enable analyses of administrative datasets that answer important clinical and health services questions). You comment on this in the conclusion, but the rationale for conversion might be more helpful right at the beginning.

Response: Thank you for your comment. We have revised the abstract – background section according to the reviewer’s comment (Abstract, page 2, lines 28 - 31):

*“A classification system that categorizes International Statistical Classification of Diseases and Related Health Problems, Tenth Revision (ICD-10) diagnosis codes into clinically meaningful categories is important for pediatric clinical and health services research using administrative data.”*

**R1.2.1** Introduction - Again, would be helpful to take some space here to explain that one cannot meaningfully analyze administrative datasets without such groupers because analyses cannot accommodate/run when several thousand diagnosis codes are included. By grouping them, we are able to ask important research questions using advanced observational study designs.

Response: Thank you for your comment. We have now added a few sentences in the Introduction section indicating the importance and use of having classification systems that group diagnosis codes into clinically relevant categories (Introduction, page 4, lines 67 - 74):

*“The thousands of specific International Statistical Classification of Diseases and Related Health Problems, Tenth Revision, Canada (ICD-10-CA) diagnosis codes present, make it difficult to meaningfully analyze administrative data without using classification systems that map the specific codes into clinically relevant categories (e.g. pneumonia, depression). By grouping the diagnosis codes, researchers, payers, or policy makers can examine patterns in healthcare utilization and costs, develop patient cohorts for research, and answer important research questions using advanced observational study designs.”*

**R1.2.2** Introduction - It would also be helpful to provide some brief literature review here of examples of using PECCS or CCS to define high-priority conditions or answer helpful clinical research questions.

Response: Thank you for your comment. We have now revised the Introduction section and have cited a few studies that have used PECCS in the United States. Two studies that were cited used PECCS to classify diagnosis of pediatric inpatient hospital encounters into mutually exclusive condition categories to identify high priority conditions (based on prevalence and cost) in children’s hospitals exclusively and another using data from both children’s and general hospitals. We also included one study that identified high priority conditions in children with ambulatory surgery hospital encounters, and another study that used PECCS to classify the diverse comorbidities present among pediatric patients admitted with catatonia (Introduction, page 5, lines 97 - 102):

*“In the US, PECCS has been used in studies to group diagnosis codes of pediatric hospital encounters into mutually exclusive condition categories in children’s hospitals exclusively, [12] and in general and children’s hospitals [13] to identify high priority conditions based on prevalence and costs. It has also been used to identify high priority conditions in pediatric ambulatory surgeries[14], and to classify the comorbidities present in pediatric patients hospitalized with catatonia [15].”*

**R1.2.3** Introduction - Need to add some background on the other classification system options available and their limitations- why do we need PECCS-CA?

Response: Thank you for your comment. We have now revised the Introduction section to add some background on the other classification system options available and why we need PECCS-CA (Introduction, pages 4 – 5, lines 76 - 88):

*“There are a few existing groupers that categorize ICD-10-CA diagnosis codes into clinical categories. One of these grouping methodologies is a translation of the Clinical Classifications Software (CCS) from the United States (US) ICD-10 codes (i.e. ICD-10-CM [Clinical Modification]) to the Canadian codes.[3] In this grouping methodology, the ICD-10-CA codes were mapped to 130 clinical categories of chronic health conditions.[3] Other grouping methodologies include the ICD-10-CA chapters which classifies diseases and related health problems and contains 23 broad category chapters,[4] and the CIHI Case Mix Group (CMG) which categorizes acute care inpatients into clinically relevant groups using diagnosis and intervention codes from the patient’s hospital record.[5,6] However, limitations in these grouping methodologies such as only categorizing diagnosis codes into chronic health condition categories, or lacking important pediatric conditions prevent its’ use in pediatric health services research. To date, a classification system that categorizes ICD-10-CA diagnosis codes in health administrative data into pediatric specific, mutually exclusive categories does not exist.”*

**R1.3** Methods - Would be helpful to provide brief descriptions of the data sources and elements contained in each as well as how they are linked and how data quality is monitored/maintained.

Response: Thank you for your comment. We have now added some details on the data sources and elements contained within the data sources, how the data is linked at ICES, and how the data quality is monitored (Methods, page 8, lines 161 - 172):

*“Datasets at ICES are linked using unique encoded identifiers known as the confidential ICES Key Number (IKN). ICES contains policies and procedures for its’ data handling practices, and every three years these policies are reviewed and approved by the Office of the Information Privacy Commissioner.[17] At ICES, a set of data standardization rules are applied to all datasets, data cleaning is conducted, the quality of data is routinely assessed and documented using the ICES’ Data Quality Framework for five different dimensions (Accuracy, Internal validity, External validity, Interpretability, and Relevance), and information about the data (e.g. data quality reports, how the data is collected) are held in an internal website on the ICES Intranet. In this study the Canadian Institute for Health Information Discharge Abstract Database (CIHI-DAD) and Same Day Surgery (SDS) database were utilized to obtain data on the inpatient and same day surgery hospital encounters including the admission date, age at admission, and the main responsible diagnosis recorded for the encounter using ICD-10-CA codes.”*

**R1.4** Results - The top 10 rankings seem a bit surprising and different from other prior similar analyses. Although this may be out of the scope of this analysis, it would be helpful to directly compare the results of using this grouper PECCS-CA vs CCS using the same dataset, or at least qualitatively compare the findings to such prior analyses. That would allow some more in depth assessment of the validity/success of the authors mapping process as well as potentially enable authors to directly demonstrate the advantages of this new classification system. Those would both be valuable additions to both the results and discussion.

Response: Thank you for your comment. The few studies that have conducted similar analyses identifying the top prevalent conditions were conducted using inpatient pediatric hospital encounters only, thus, making it difficult to conduct a direct comparison to our study, which additionally used ambulatory surgery encounters. However, one US study conducted by Keren *et al.* (2012)[10] focused on children with hospital encounters (included inpatient and ambulatory surgery) also found otitis media, hypertrophy of tonsils and adenoids, bronchiolitis, pneumonia, and dental caries within the top 10 most prevalent conditions. Although, as the reviewer mentioned, it would be helpful to directly compare the results of using PECCS-CA vs CCS using the same dataset, it is difficult to conduct such analysis. The CCS is a grouping methodology that is used to categorize the US ICD-10 codes (i.e. ICD-10-CM), while the PECCS-CA is used to categorize the Canadian version of the ICD-10 codes (i.e. ICD-10-CA). Furthermore, there are no studies that have used CCS to identify and rank the most prevalent conditions in children with hospital encounters, regardless of the diagnosis, to compare it to our current study. Nevertheless, our research letter published on the PECCS for the US ICD-10-CM codes compared the detection of conditions in PECCS versus CCS using data from children with hospital encounters from the US children’s hospitals. We showed that there was an increased specificity of detecting pediatric health conditions using PECCS compared to the CCS. Please see Gill *et al.* (2021)[7] for the comparison between PECCS and CCS. If the CCS was able to be used in our current study to identify the most prevalent conditions in children, important pediatric conditions such as bronchiolitis, neonatal hyperbilirubinemia, and transient tachypnea of newborn would have been missed. We have revised the discussion section to include the above mentioned points (Discussion, pages 17-18, lines 285 - 300):

*“The CCS is a grouper used to classify the ICD-10-CM codes into clinically meaningful categories,[9] and the beta version (2019.1) of the CCS was used to develop the original PECCS for the ICD-10-CM codes.[7,8] The beta version of the CCS categorized more than 70,000 ICD-10-CM diagnosis codes into 283 clinical categories.[21] The utility of PECCS-CA was not compared to the CCS using the same hospital encounter dataset due to the differences in the ICD-10 coding systems. However, we previously compared the original PECCS’s ability to detect pediatric conditions with the CCS using pediatric hospitalization data from the US.[7] The PECCS demonstrated increased specificity of detecting pediatric health conditions. For instance, 13,261 pediatric hospital encounters in the US were classified into miscellaneous mental health disorders using CCS, while the same encounters were classified into the following when PECCS was used: miscellaneous mental health disorders (5,357 encounters), anorexia nervosa (4,709 encounters), conversion disorder (2,979 encounters), and bulimia nervosa (216 encounters).[7] If the CCS was able to be applied to our current dataset, important pediatric conditions including bronchiolitis, neonatal hyperbilirubinemia, and transient tachypnea of newborn would not have been detected. This further demonstrates the importance of PECCS-CA for pediatric health services research in Canada.”*

***REVIEWER #2:***

**R2.1** This cross-sectional study used a rational, stepwise approach to map ICD-10-CA codes onto ICD-10-CM codes to adapt the US-based PECCS classification system to Canadian settings. The study is an important contribution to pediatric health services research since it will allow for research that is specific to child health in Canada. The authors’ newly developed PECCS-CA classification system will be publicly available, which will be very helpful to pediatric health services researchers tracking healthcare utilization, cost, and outcomes in Canada. I have no major or minor concerns about this manuscript – it is clearly written and the discussion does a good job providing context for why development of this pediatric-specific and country-specific classification system is important.

Although authors are not able to make the data used for this research publicly available, they provide a reasonable answer for why this is the case.

Response: Thank you very much for your comments.

**RC1.6** PLOS authors have the option to publish the peer review history of their article ([what does this mean?](https://urldefense.com/v3/__https:/journals.plos.org/plosone/s/editorial-and-peer-review-process*loc-peer-review-history__;Iw!!D0zGoin7BXfl!-MRa53UALKDUf89OUVuV6G6Wm5BCskJ4Gumovk4h853FnskRx-Xi-5elo572tEv_wYoAdm9Q0aAOoAlPDWPzlko_$)). If published, this will include your full peer review and any attached files.

If you choose “no”, your identity will remain anonymous but your review may still be made public.

**Do you want your identity to be public for this peer review?** For information about this choice, including consent withdrawal, please see our [Privacy Policy](https://urldefense.com/v3/__https:/www.plos.org/privacy-policy__;!!D0zGoin7BXfl!-MRa53UALKDUf89OUVuV6G6Wm5BCskJ4Gumovk4h853FnskRx-Xi-5elo572tEv_wYoAdm9Q0aAOoAlPDc4GD0RQ$).

Reviewer #1: No

Reviewer #2: No

Response: Thank you for your response.

Thank you for taking the time to consider our revised manuscript. Please let us know if you need any other additional information.

Sincerely,

| **Peter J Gill MSc DPhil MD FRCPC**  Staff Paediatrician, Division of Paediatric Medicine  Assistant Professor, Dept of Paediatrics, Univ of Toronto  Associate Scientist, SickKids Research Institute  The Hospital for Sick Children  Phone: 416-813-8525  Email: [peter.gill@sickkids.ca](mailto:peter.gill@sickkids.ca) | **Sanjay Mahant** **MD MSc FRCPC**  Staff Paediatrician, Division of Paediatric Medicine  Professor, Dept of Paediatrics, Univ of Toronto  Associate Scientist, SickKids Research Institute  The Hospital for Sick Children  Phone: 416-813-5280  Email: [sanjay.mahant@sickkids.ca](mailto:sanjay.mahant@sickkids.ca) |
| --- | --- |
